# Astaxanthin From *Haematococcus pluvialis* Prevents High-Fat Diet-Induced Hepatic Steatosis and Oxidative Stress in Mice by Gut-Liver Axis Modulating Properties

**DOI:** 10.3389/fnut.2022.840648

**Published:** 2022-04-12

**Authors:** Meng Wang, Wenxin Xu, Jie Yu, Yingying Liu, Haotian Ma, Chunli Ji, Chunhui Zhang, Jinai Xue, Runzhi Li, Hongli Cui

**Affiliations:** ^1^College of Agriculture, Institute of Molecular Agriculture and Bioenergy, Shanxi Agricultural University, Jinzhong, China; ^2^Health Science Center, College of Forensic Sciences, Xi’an Jiaotong University, Xi’an, China; ^3^State Key Laboratory of Integrative Sustainable Dryland Agriculture, Shanxi Agricultural University, Taiyuan, China

**Keywords:** astaxanthin, oxidative stress, hepatic steatosis, lipid metabolism, gut microbiota

## Abstract

**Scope:**

Evidence is mounting that astaxanthin (ATX), a xanthophyll carotenoid, used as a nutritional supplement to prevent chronic metabolic diseases. The present study aims to identify the potential function of ATX supplementation in preventing steatohepatitis and hepatic oxidative stress in diet-induced obese mice.

**Methods and Results:**

In this study, ATX as dose of 0.25, 0.5, and 0.75% have orally administered to mice along with a high-fat diet (HFD) to investigate the role of ATX in regulating liver lipid metabolism and gut microbiota. The study showed that ATX dose-dependently reduces body weight, lipid droplet formation, hepatic triglycerides and ameliorated hepatic steatosis and oxidative stress. 0.75% ATX altered the levels of 34 lipid metabolites related to hepatic cholesterol and fatty acid metabolism which might be associated with downregulation of lipogenesis-related genes and upregulation of bile acid biosynthesis-related genes. The result also revealed that ATX alleviates HFD-induced gut microbiota dysbiosis by significantly inhibiting the growth of obesity-related *Parabacteroides* and *Desulfovibrio* while promoting the growth of *Allobaculum* and *Akkermansia*.

**Conclusion:**

The study results suggested that dietary ATX may prevent the development of hepatic steatosis and oxidative stress with the risk of metabolic disease by gut-liver axis modulating properties.

## Graphical Abstract

Astaxanthin (ATX) preventive effects on lipid metabolic disorders, hepatic steatosis, and oxidative stress in high-fat diet-induced mice.

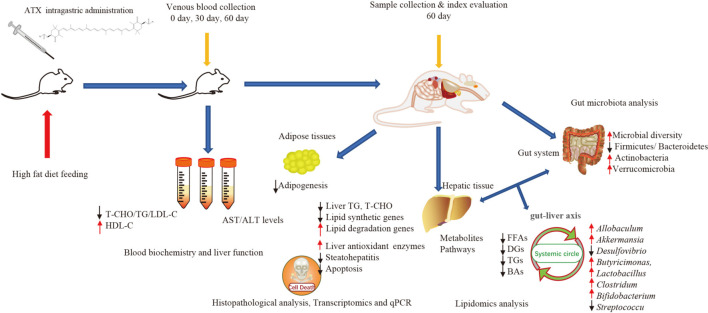

## Introduction

Over the past few decades, obesity has become globally recognized as one of the most common life-threatening chronic metabolic problems, with steadily increasing rates year by year, which are ascribed to the imbalance of energy metabolism and excessive fat accumulation in the body (1, 2). Obesity causes a series of abnormal metabolic complications, including lipid metabolism, oxidant stress, inflammatory responses, insulin resistance (IR) and steatohepatitis (3, 4). Notably, among patients with NAFLD, obese individuals present more severe histological phenotypes and may suffer from higher mortality and morbidity (5). Recently, according to an epidemiological survey on nutrition and health, the prevalence of obesity has gradually become appeared in younger individuals (6). In modern society, a suboptimal diet and little exercise are among the leading causes of poor health, also increasing triacylglycerol (TG), total cholesterol (TC), free fatty acid (FFA) accumulation, and lipid peroxidation (7, 8). For example, systemic oxidative stress, a key factor in pathological obesity, can be induced by a high-calorie diet through various mechanisms (9, 10).

Currently, the existing anti-obesity medications that have been developed are unsuitable for certain individuals with obesity due to potential side effects and drug tolerability (11, 12). There is, therefore, a key practical value to search for safe and effective functional components from natural foods to prevent obesity and related metabolic diseases when compared with synthetic drugs. With health benefits increasing in the human diet, carotenoids, natural antioxidants distributed in numerous microbes, plants, and animals, have received considerable scholarly attention in recent years (13). Furthermore, an unambiguous association was found between the mechanism of carotenoids regulating liver lipid metabolism and the incidence of obesity-related NAFLD, as demonstrated in many studies (13–15).

Astaxanthin (ATX extracted from *Haematococcus pluvialis*) is a xanthophyll carotenoid in marine organisms and is often used as a nutritious supplementary food in the daily diet. ATX has a protective effect against oxidative stress, inflammation, and metabolic disorders, such as liver fibrosis and type 2 diabetes (16, 17). In our previous study, after 12 weeks of high-fat intake, we found that C57BL/6 mice exhibited a significant reduction in serum TG and TC by administering intragastric ATX treatment (18). Furthermore, ATX also improved lipid metabolism by regulating lipid-related gene and metabolite contents. According to many previous animal experiments, consumption of ATX not only showed no signs of poisoning but also exerted a positive pharmacological effect (19, 20).

Recently, the relationship between gut microbiota and metabolic diseases has attracted attention from the scholarly community because intestinal flora has been confirmed as a target for the prevention and treatment of obesity, metabolic syndrome and cardiovascular diseases (21). Therefore, it is important to investigate how ATX prevents the development of hepatic steatosis and oxidative stress with the risk of metabolic disease. The present study aims to contribute to this growing area of research by exploring the prevention effects on obesity and the development of NAFLD through long-term dietary ATX in mice.

## Materials and Methods

### Chemicals

The detection kits for alanine transaminase (ALT), aspartate transaminase (AST), TG, TC, high-density lipoprotein cholesterol (HDL-C), and low-density lipoprotein cholesterol (LDL-C), and the antioxidant assay kits for SOD, CAT, GSH, T-AOC, and malondialdehyde (MDA) were purchased from Nanjing Jiancheng Bioengineering Institute (Nanjing, China). The superoxide indicator dihydroethidium ROS probe (DHE), 4’,6-diamidino-2-phenylindole (DAPI) and YF488 TUNEL assay apoptosis detection kit were purchased from US Everbright, Inc. Other chemicals, solvents and reagents used in the present study were of laboratory analytical grade.

### Preparation of Astaxanthin

Astaxanthin oleoresin was provided by Shandong Jinjing Biotechnology Co., Ltd., and the purification was performed in our lab during a previous study (18). After purification, ATX of 94.69% purity was used in the current study.

### Animals

The Institutional Animal Care and Use Committee of Shanxi Agricultural University approved all experimental protocols for animal care, handling and experimentation (SXAU-EAW-2018-112). We also confirmed that all experiments were conducted in accordance with relevant guidelines and regulations. Forty-eight male C57BL/6 mice (SPF, 8 weeks old) weighing 20–22 g were obtained from the Experimental Animal Center of Shanxi Medical University. They were housed in individual cages in a specific room (22 ± 2°C) with 55% humidity on a 12 h dark/light schedule and had free access to food and pure water at the Institute of Molecular Agriculture and Bioenergy, Shanxi Agriculture University.

### Dosage Information

The design of animal experiments was based on our previous methods (18). After a 2-week acclimation period, mice were randomly assigned to six groups (each comprising eight mice): the normal diet group (ND), the high-fat diet (HFD) group, the solvent group, the HFD + 0.25% ATX group, the HFD + 0.5% ATX group, and the HFD + 0.75% ATX group. The mice in the ND group were fed standard rodent chow containing 3.5 kcal/g with 4.3% fat, 67.2% carbohydrates, and 19.2% protein. The mice in the other group were fed a HFD containing 4.73 kcal/g with 24% fat, 41% carbohydrates, and 24% protein. The mice in the ND group were given distilled water, and the solvent group was gavaged with corn oil. In addition, the mice in the ATX treatment groups were gavaged with 0.25, 0.5, and 0.75% ATX dissolved in corn oil. During the diet phase, all mice were given intragastric treatment once per day at 9:00 a.m., which lasted 9 weeks. The diets were purchased from Beijing Huafukang Bioscience Co., Ltd. Supplementary Table 1 shows the ingredients of the experimental diets.

### Physiological Evaluation

The body weight and food intake were recorded daily for 63 days. To avoid error values, the measurement of weight was repeated three times for each mouse. The energy intake was calculated as food intake × 4.73 kcal g^–1^ for the HFD group and food intake × 3.50 kcal g^–1^ for the ND group, as demonstrated in previously (18). Mice were fasted for 12 h after the last treatment and then euthanized by inhalation with isoflurane. Blood samples were obtained from the retro-orbital veins on Days 0, 30, and 60. The serum was separated by centrifugation at 3,000 rpm for 15 min at 4°C and stored at −20°C until analysis. All other organs, including the liver, heart, kidney, spleen, and adipose tissues, were immediately collected and weighed individually after sacrificing the animals.

### Biochemical Analysis

The serum TG, TC, HDL-C, and LDL-C levels and activities of AST (GOT) and ALT were determined using biochemical kits according to the standards and protocols provided by the manufacturer (Nanjing, China). According to the ratio of weight (g): volume (mL) = 1:9, the liver tissues were homogenized with phosphate buffer (PH 7.4) at 4°C and centrifuged at 2,500 rpm for 10 min at 4°C. The supernatant was collected to determine the protein and lipid levels (TG and TC) and enzymatic analyses (T-AOC, SOD, CAT, GSH, MDA, and ROS).

### Histopathological Analysis

The dihydroethidium (DHE) probe method was used to qualitatively detect ROS. Five-micron-thick sections of the liver were dyed with DHE, and incubation was performed at 37°C for 10 min in a dark environment. The samples were directly observed under a fluorescence microscope at a measuring emission of 595 nm. The ROS-positive cells had strong red fluorescence. After sacrificing each mouse, fresh epididymal adipose tissue (e-AT) and liver tissues were immersed in 4% paraformaldehyde for 24 h. The tissues were embedded in paraffin, sectioned at a 5 μm thickness and stained with H&E. Meanwhile, the frozen sections were stained with Oil Red O (ORO), which was performed to further detect hepatic vacuolization, inflammatory cell infiltration, and lipid droplets. All of the samples were photographed under a light microscope (Leica, Germany) at 20×/40× magnification.

The above sections were used to examine hepatocellular apoptosis with the YF488 TUNEL assay apoptosis detection kit. After the TUNEL reaction, the sections were mounted using antifade mounting medium with DAPI and observed under an inverted fluorescence microscope at 385 and 485 nm wavelength excitation. The negative cells were dyed with blue fluorescence intensity at 385 nm, while the apoptotic cells exhibited green fluorescence at 485 nm. ImageJ software (National Institutes of Health, United States) was used to measure the cell counting of sections from each group.


$$\mathrm{The}\mathrm{apoptosis}\mathrm{rate}\left(\%\right)=\frac{\mathrm{Apoptotic}\,\mathrm{cell}\,\mathrm{number}}{\mathrm{Total}\,\mathrm{cell}\,\mathrm{number}}\times 100$$

### Polymerase Chain Reaction Analysis

Total RNA was extracted from liver tissue using TRIzol reagent (Shenggong BBI Life, Shanghai, China) according to the manufacturer’s instructions. Then, cDNA was synthesized from total RNA using the PrimeScript Reverse Transcription reagent kit (Takara, Dalian, China). Quantitative polymerase chain reaction (PCR) was conducted in triplicate for each group to detect gene expression. The quantitative analysis of *AMPK*, *SREBP1c*, *ACC*, *CPT-1*, *PPARα*, *PPARγ*, *LXRα*, *SCD-1*, *PGC-1*, *FAS*, *CYP27A1*, and *CYP7A1* mRNA expression in the liver was measured in triplicate for each group by quantitative PCR. According to the SYBR Premix Ex Taq II (Takara, Dalian, China), the thermal cycle of qPCR was reacted on the CFX 96 Real-Time PCR Detection system (BIO-RAD, Hercules, CA, United States) under the following conditions: 95°C for 10 min, then 40 cycles of 95°C for 15 s, 60°C for 30 s, and 72°C for 30 s. Supplementary Table 2 shows the PCR primer sequences of each gene, and the target genes were normalized to the reference gene GAPDH. The 2^–ΔΔCt^ method was used to calculate relative gene expression.

### Transcriptomics and Lipidomics Analysis

To investigate lipid metabolism, fresh samples were sent to MetWare Biotechnology Co., Ltd., Wuhan, to apply transcriptomics and metabolomics analyses. The extraction of liver lipids and lipidomic analysis were performed using an LC–MS/MS system following previous works (18). After RNA was extracted from liver biopsy samples, liver transcriptome analysis was conducted by RNA sequencing, as described in detail previously (22, 23).

### 16S rRNA Sequencing Analysis

The caecal contents were sent to Shanghai Personal Biotechnology Co., Ltd. to investigate microbial diversity through 16S rRNA analysis on the Illumina MiSeq platform. When the microbial DNA was isolated, PCR of the V3–V4 region of the bacterial 16S rRNA gene was performed using the forward primer 5′-ACTCCTACGGGAGGCAGCA-3′ and the reverse primer 5′-GGACTACHVGGGTWTCTAAT-3′ according to the manufacturer’s protocol (24, 25). A previous study illustrated the analytical conditions and detailed parameters (18).

### Statistical Analysis

All experiments were biologically repeated three times, and the data were analyzed with Social Sciences (SPSS 16.0) statistical software and are presented as the mean ± SD. Multiple comparisons among treatments were statistically analyzed using Duncan’s multiple range test in one-way analysis of variance (ANOVA) (*P* < 0.05). Origin 9.1 was used to draw charts.

## Results

### Effect of Astaxanthin Supplementation on Body Weight and Calorie Intake

**FIGURE 1:**
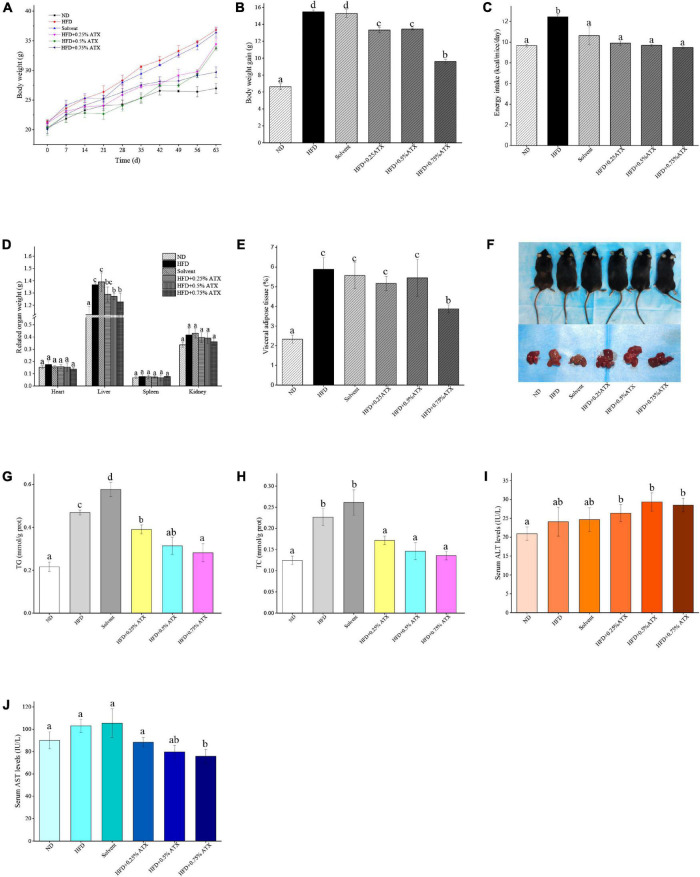
Astaxanthin (ATX) prevented obesity and related indices in HFD-fed mice. **(A)** Body weight. **(B)** Body weight gain. **(C)** Energy intake. **(D)** Related organ weight. **(E)** Visceral adipose tissue. **(F)** Visual appearance pictures of metabolic mice and liver. **(G)** Hepatic TG level. **(H)** Hepatic TC level. **(I)** Serum ALT level. **(J)** Serum AST level. Values are expressed as mean ± SD of triplicate (*n* = 6). The different letters (a–d) indicate significant differences (*P* < 0.05) according to Duncan’s multiple range test in ANOVA.

A dramatic increment in body weight was observed in the HFD group, while a moderate increase was observed in the ATX treatment groups (Figure 1A). Body weight gain plays a pivotal role in evaluating the effect of HFD on obesity and assessing its prevention. Table 1 presents the initial and final body weights of mice in each group. During feeding induction, the mice gained more weight in the HFD group (15.48 ± 0.22 g) and the solvent group (15.29 ± 0.46 g) than in the ND group (6.62 ± 0.34 g). Meanwhile, ATX supplementation in mice decreased weight gain, and a high dose of ATX significantly (*P* < 0.05) lowered body weight gain (9.60 ± 0.28 g) compared to HFD alone (Figure 1B). The energy intake of mice in the HFD group and the solvent group were significantly (*P* < 0.05) higher than the ND group; however, ATX consumption could reduce the energy intake compared to the HFD group regardless of the doses (Figure 1C). There was no significant difference in the food efficiency ratio of the mice in each group except for the ND group (Table 1).

**TABLE 1:** Effect of astaxanthin (ATX) supplementation on body weight, energy intake, and food efficiency ratio in high-fat diet-induced mice*^a^*.

Group	ND	HFD	Solvent	HFD+0.25% ATX	HFD+0.5% ATX	HFD+0.75% ATX
Initial body weight (g/mice)	20.35 ± 0.14^a^	21.35 ± 0.35^a^	21.09 ± 0.50^a^	21.11 ± 0.37^a^	20.32 ± 1.26^a^	20.08 ± 0.71^a^
Final body weight (g/mice)	26.97 ± 0.87^a^	36.83 ± 0.47^d^	36.38 ± 0.75^d^	34.79 ± 0.96^dc^	33.76 ± 0.34^c^	29.39 ± 0.88^b^
Weight gain (g/mice)	6.62 ± 0.34^a^	15.48 ± 0.22^d^	15.29 ± 0.46^d^	13.32 ± 0.33^c^	13.44 ± 0.12^c^	9.60 ± 0.28^b^
Energy intake (kcal/mice/day)	9.66 ± 0.14^a^	12.46 ± 0.19^b^	10.63 ± 0.85^a^	9.90 ± 0.21^a^	9.69 ± 0.12^a^	9.47 ± 0.08^a^
Food efficiency ratio (%)^b^	0.47 ± 0.15^a^	1.05 ± 0.28^b^	1.36 ± 0.41^b^	1.06 ± 0.29^b^	1.06 ± 0.30^b^	0.96 ± 0.21^b^

*^a^The body weight, energy intake and food efficiency ratio of mice in each group were tested based on one-way ANOVA and the results are expressed as the mean ± SD (n = 6). ^b^Food efficiency ratio (%) = (weekly weight gain / weekly food intake) × 100. The values with different labels (a–d) in rows refer to significant difference (P < 0.05).*

### Effect of Astaxanthin on Wet Liver Weights of and Fat Droplet Deposition

To estimate whether the HFD with ATX supplementation affected visceral organs and fat, the wet weights of adipose tissue and organs were measured in each group, especially the mouse liver (Figure 1D). There were no significant differences in the heart, spleen or kidney in each group, similar to our previous results (18) (Table 2). Compared to the ND group, the wet liver weights and fat were significantly (*P* < 0.05) increased in the HFD group. ATX supplementation (0.5% and 0.75% w/w) significantly (*P* < 0.05) decreased the wet weights of the liver in high-fat diet-fed mice. In addition, visual adipose tissue, including perirenal and epididymal adipose tissue, was significantly (*P* < 0.05) lowered in the 0.75% ATX group compared to the HFD group (Figure 1E).

**TABLE 2:** Effect of ATX supplementation on related organ weights and adipose tissue weights in high-fat diet-induced mice*^a^*.

Group	ND	HFD	Solvent	HFD+0.25% ATX	HFD+0.5% ATX	HFD+0.75% ATX
Heart (g)	0.15 ± 0.01^a^	0.18 ± 0.03^a^	0.16 ± 0.01^a^	0.16 ± 0.02^a^	0.16 ± 0.03^a^	0.14 ± 0.01^a^
Liver (g)	1.12 ± 0.07^a^	1.37 ± 0.02^c^	1.39 ± 0.08^c^	1.29 ± 0.06^bc^	1.27 ± 0.03^b^	1.23 ± 0.07^b^
Spleen (g)	0.07 ± 0.01^a^	0.08 ± 0.02^a^	0.08 ± 0.02^a^	0.07 ± 0.01^a^	0.07 ± 0.02^a^	0.08 ± 0.01^a^
Kidney (g)	0.34 ± 0.02^a^	0.42 ± 0.05^a^	0.43 ± 0.04^a^	0.40 ± 0.04^a^	0.39 ± 0.04^a^	0.36 ± 0.03^a^
Visceral adipose tissue (g)	0.63 ± 0.20^a^	2.17 ± 0.60^c^	2.03 ± 0.66^c^	1.84 ± 0.34^c^	1.78 ± 0.37^c^	1.15 ± 0.20^b^

*^a^All values are expressed as the mean ± SD (n = 6). Mean separation was performed using Duncan’s multiple range test. The different letters (a–c) indicate significant differences (P < 0.05). The visceral adipose tissue included perirenal adipose and epididymal adipose.*

Liver lipid indicators, namely TG and TC levels, are important parameters for obtaining an understanding of diet-induced fat deposition. The liver turned brown following the accumulation of TG and TC in the HFD group, indicating dyslipidaemia, possibly leading to other diseases. This phenomenon was suppressed in the ATX treatment group compared to the HFD group (Figure 1F). The TG and TC levels were examined to further quantify liver fat deposition, as shown in Figures 1G,H. The TG and TC levels were significantly (*P* < 0.05) increased by 117.4 and 82% in the HFD group compared to the ND group. However, ATX supplementation effectively decreased fat deposition in a dose-dependent manner compared to the HFD group, among which the TG and TC levels were 40.1 and 40%, respectively.

### Effect of Astaxanthin on the Serum Lipid Profiles and Liver Function Indicators of High-Fat Diet-Induced Mice

Table 3 presents the serum lipid profiles of mice at 0, 30, and 60 days. There were no obvious differences in the initial serum lipid profiles among the six groups. The TG, TC, and LDL-C levels of mice were significantly (*P* < 0.05) increased, while the HDL-C level was reduced in the HFD and the solvent groups after 30 and 60 days. Such results indicated that lipid metabolism was disordered. The TC, TG, and LDL-C levels of mice were significantly (*P* < 0.05) decreased in the ATX treatment groups compared to the HFD group after 60 days despite the indistinctive (*P* > 0.05) change on Day 30. The mice in the 0.75% ATX group showed slightly higher serum HDL-C levels than the mice fed a HFD alone, but the variation was not significant on Day 30 (*P* > 0.05). When compared with the HFD group, serum TG levels in the 0.5 and 0.75% ATX groups were significantly decreased by 16.9 and 21.7% on Day 60 (*P* < 0.05), respectively. Serum TC levels in the 0.25, 0.5 and 0.75% ATX groups were significantly reduced to 3.65 ± 0.18, 3.16 ± 0.09, and 2.94 ± 0.38 mmol/L on Day 60 (*P* < 0.05), respectively. Serum LDL-C levels in the 0.5 and 0.75% ATX groups exhibited significant reductions of 35.2 and 39.6% (*P* < 0.05), respectively. No apparent changes were monitored after supplementing ATX compared with HFD feeding alone (*P* > 0.05). Therefore, based on the results mentioned above, 0.5 and 0.75% ATX supplementation could efficiently reduce the levels of TG and TC.

**TABLE 3:** Effect of ATX supplementation on the levels of serum TG, TC, HDL-C, and LDL-C in the HFD-fed mice.

	ND	HFD	Solvent	HFD+0.25% ATX	HFD+0.5% ATX	HFD+0.75% ATX
**TG (mmol/L)**						
0 day	0.41 ± 0.09^a^	0.43 ± 0.18^a^	0.45 ± 0.28^a^	0.44 ± 0.23^a^	0.43 ± 0.10^a^	0.42 ± 0.11^a^
30 days	0.48 ± 0.08^a^	0.72 ± 0.07^b^	0.70 ± 0.14^ab^	0.64 ± 0.05^ab^	0.54 ± 0.08^ab^	0.45 ± 0.18^ab^
60 days	0.47 ± 0.13^a^	0.83 ± 0.12^c^	0.78 ± 0.21^ab^	0.74 ± 0.10^b^	0.69 ± 0.15^ab^	0.65 ± 0.14^ab^
**TC (mmol/L)**						
0 day	3.40 ± 0.28^a^	3.49 ± 0.10^a^	3.47 ± 0.13^a^	3.36 ± 0.23^a^	3.38 ± 0.18^a^	3.30 ± 0.10^a^
30 days	3.36 ± 0.08^a^	4.19 ± 0.49^b^	4.08 ± 0.34^b^	3.56 ± 0.33^ab^	3.29 ± 0.30^a^	3.26 ± 0.57^a^
60 days	3.39 ± 0.12^a^	4.73 ± 0.38^b^	4.35 ± 0.41^b^	3.65 ± 0.18^a^	3.16 ± 0.09^a^	2.94 ± 0.38^a^
**HDL-C (mmol/L)**						
0 day	0.59 ± 0.08^a^	0.60 ± 0.06^a^	0.62 ± 0.12^a^	0.60 ± 0.14^a^	0.62 ± 0.09^a^	0.56 ± 0.08^a^
30 days	0.49 ± 0.04^a^	0.36 ± 0.02^b^	0.36 ± 0.05^b^	0.33 ± 0.03^b^	0.31 ± 0.03^b^	0.31 ± 0.08^b^
60 days	0.33 ± 0.04^a^	0.19 ± 0.07^b^	0.22 ± 0.05^b^	0.23 ± 0.04^b^	0.25 ± 0.02^b^	0.26 ± 0.04^ab^
**LDL-C (mmol/L)**						
0 day	0.36 ± 0.04^a^	0.38 ± 0.04^a^	0.37 ± 0.03^a^	0.37 ± 0.04^a^	0.38 ± 0.04^a^	0.35 ± 0.04^a^
30 days	0.39 ± 0.03^a^	0.83 ± 0.14^b^	0.74 ± 0.10^b^	0.63 ± 0.10^b^	0.51 ± 0.15^ab^	0.48 ± 0.12^ab^
60 days	0.57 ± 0.03^a^	0.91 ± 0.25^b^	0.84 ± 0.14^b^	0.70 ± 0.08^b^	0.59 ± 0.14^ab^	0.55 ± 0.12^ab^

*Data were expressed as mean ± SD of 6 mice in each group and were analyzed by ANOVA analysis with Duncan’s test. Mean values denoted with different superscript letters were significant differences (P < 0.05).*

In addition, we evaluated serum AST (GOT) and ALT to further explore the liver function induced by HFD and ATX consumption. The serum AST level was increased in the solvent and HFD groups compared with the ND group; however, the serum ALT level was not apparent in any group (Figure 1I). The mice in the 0.75% ATX group displayed significantly lower serum AST levels than the mice in the solvent group, which were reduced by 28.1% (Figure 1J).

### Effect of Astaxanthin Supplementation on Liver Levels of Oxidation Resistance Markers in High-Fat Diet-Induced Mice

**FIGURE 2:**
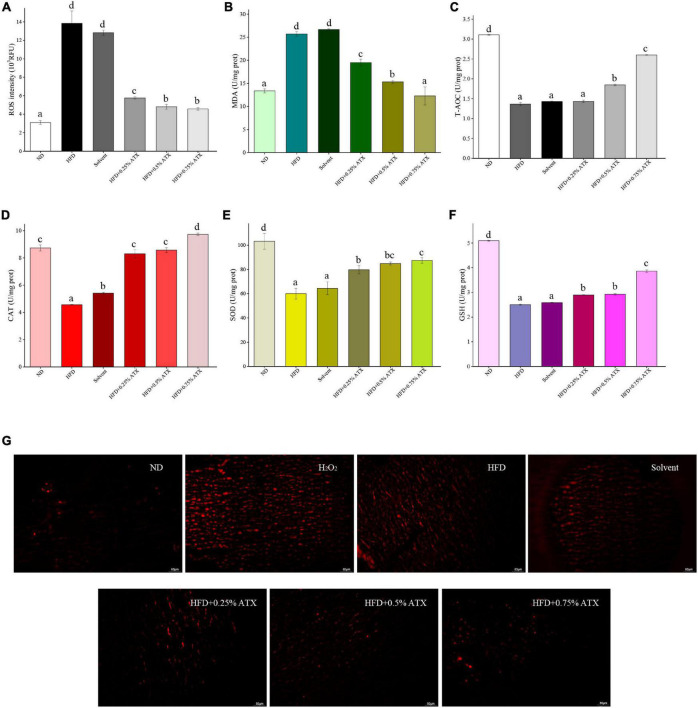
Evaluation of liver oxidation resistance in HFD-induced mice liver tissues. The levels of ROS intensity **(A)**, MDA **(B)**, T-AOC **(C)**, CAT **(D)**, SOD **(E)**, and GSH **(F)** are illustrated in the panel. Values are expressed as mean ± SD of triplicate (*n* = 6). The different letters (a–d) indicate significant differences (*P* < 0.05) according to Duncan’s multiple range test in ANOVA. **(G)** Qualitative detection of ROS by the DHE probe; the ROS-positive cells emitted bright red fluorescence; H_2_O_2_: as double positive control, and tissues were incubated with 3% H_2_O_2_.

Malondialdehyde and reactive oxygen species (ROS) activities are crucial components contributing to the development of oxidative stress in high-fat diet-fed animals. The levels of antioxidant enzymes, including T-AOC, CAT, SOD, and GSH, were assessed in the liver and exhibited similar trends. The ROS and MDA levels of mice were significantly (*P* < 0.05) increased in the HFD group compared with the ND group (Figures 2A,B). According to the ROS qualitative fluorography images, intense red fluorescence was observed in the HFD and the positive control groups (incubated with H_2_O_2_), while the faint fluorescence in the ATX-treated samples corresponded with the quantitative results (Figure 2G). The levels of T-AOC, CAT, SOD, and GSH were significantly (*P* < 0.05) decreased in the HFD group (Figures 2C–F); however, the effect of solvent on antioxidant enzymes was not significant (*P* > 0.05). Furthermore, 0.5 and 0.75% ATX supplementation effectively (*P* < 0.05) prevented lipid peroxidation in high-fat diet-fed mice, although the effect was slight in the 0.25% ATX group. When compared to the HFD group, the levels of T-AOC, CAT, SOD, and GSH were increased by 90.6, 113.2, 45.6, and 54.4% in the 0.75% ATX group, respectively.

### Histological Assessment of the Liver and Epididymal Adipose Tissue

**FIGURE 3:**
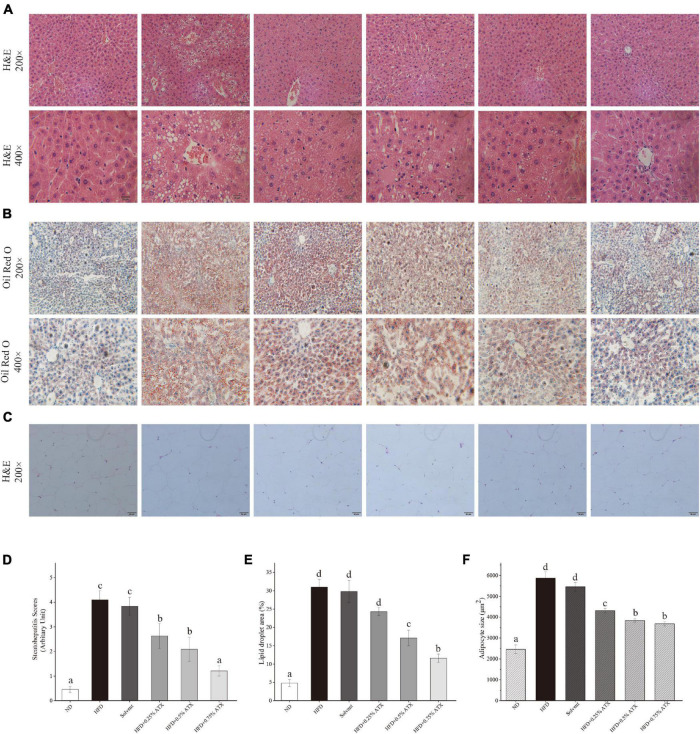
Pathological changes of ATX on liver and epididymal fat in HFD-induced mice. **(A)** Liver sections stained with HE (200×, 400×). **(B)** Liver sections stained with Oil red O (200×, 400×). **(C)** HE-stained e-AT sections (200×). **(D)** Steatohepatitis scores. **(E)** Percentage of the lipid droplet area assessed by Oil red O staining. **(F)** Mean cell area of adipocyte in e-AT. Values are expressed as mean ± SD of triplicate (*n* = 6). The different letters (a–d) indicate significant differences (*P* < 0.05) according to Duncan’s multiple range test in ANOVA.

Photomicrographs of H&E staining were used to observe the pathology in the liver tissues from all experimental groups. In the ND group mice, hepatocytes were fairly uniform, with regularly shaped hepatic plates arranged in an ordered pattern and hepatic cords, except for slight congestion (Figure 3A). However, the HFD induced typical lesions in the mouse liver, such as hepatocyte necrosis, inflammatory cell infiltration, congestion of the central veins, ballooning, hepatic sinus expansion and chromatin condensation. In the solvent group, the structure of hepatic plates was irregularly arranged along with fat accumulation, indicating that long-term excessive fat intake disturbed lipid metabolism in the liver. After ATX supplementation following HFD feeding, the pathological characteristics were prevented in the liver tissues, and the steatohepatitis scores were significantly (*P* < 0.01) lowered; in particular, the 0.75% ATX group showed a similar pattern to the ND group, implying the hepatoprotective effect of ATX (Figure 3D).

To further investigate the production of lipid droplets in the liver, Oil Red O staining was performed (Figure 3B). More oil red O-stained lipid droplets were observed in the liver tissue of the HFD and solvent groups than in the liver tissue of the ND group, resembling the percentage result of lipid droplets (Figure 3E). Conversely, ATX supplementation dose-dependently decreased the production of fatty droplets, in which the area of droplets was significantly lessened in the 0.5 and 0.75% ATX groups. These results confirmed that ATX prevented lipid accumulation and hepatic steatosis, conforming to the results of intrahepatic TG and TC levels. As shown in the e-AT sections of HFD-induced mice (Figures 3C,F), the mean adipocyte size increased almost 138.43% compared with that of mice in the ND group. These results were markedly (*P* < 0.05, Table 2) related with increasing visual adipose tissue weight.

Apoptotic cells were detected by green fluorescent TUNEL staining, and cell nuclei were stained blue (DAPI). The results showed that the positive apoptosis rate significantly (*P* < 0.05) increased along with the long-term fat infiltration in liver tissue caused by HFD (Supplementary Figure 1A). Compared to that in the HFD group, the number of apoptotic cells stained green was reduced in a dose-dependent manner with ATX supplementation, and the apoptosis rates were decreased by 14.7, 25.1, and 48.5%, respectively (Supplementary Figure 1B).

### Effect of Astaxanthin Supplementation on the Relative mRNA Expression Levels and Transcriptome Level Verification

**FIGURE 4:**
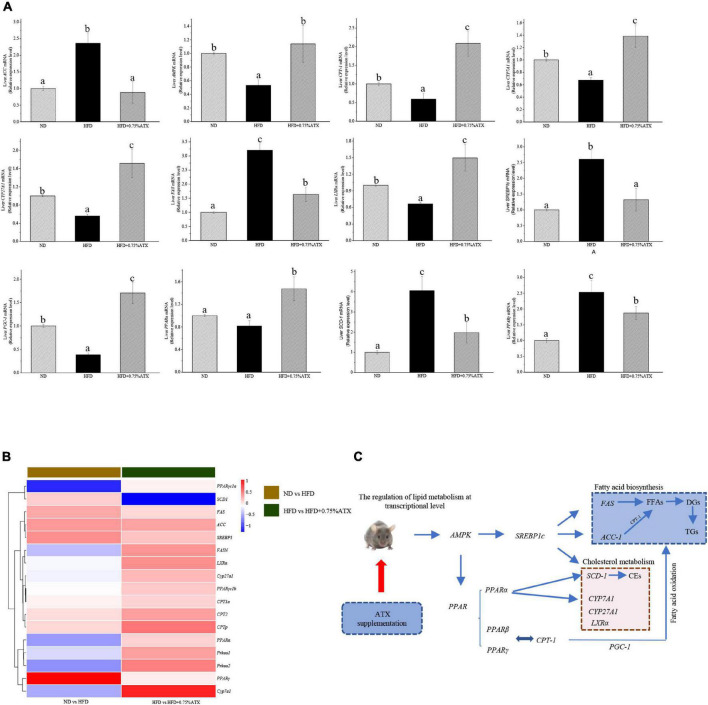
Astaxanthin significantly improved relative gene expression. **(A)** Gene expression levels in AMPK/SREBP1c pathway. **(B)** The heatmap of differential genes expression at the transcriptional level. **(C)** Regulatory effects of ATX supplementation on fatty acid and cholesterol metabolism in mice induced by HFD. Data are shown as mean ± SD of triplicate. The different letters (a–c) indicate significant differences (*P <* 0.05) according to Duncan’s multiple range test in ANOVA.

To understand the mechanism(s) by which ATX modulates hepatic lipid metabolism in response to a high-fat diet, we analyzed the expression of genes related to lipogenesis and fatty acid β-oxidation in the liver by qRT–PCR. As shown in Figure 4A, the expression of *SREBP1c* in the livers of HFD group mice significantly (*P* < 0.05) increased and the expression of *AMPK* and *PPARα* decreased compared to those in ND group mice. However, the expression levels of these key genes were notably altered after ATX supplementation, although no significant differences in the mRNA levels of *AMPK* and *SREBP1c*, the key factors of metabolic energy and transcription factors in lipogenesis, were visualized in either the ND or HFD + 0.75% ATX group. The expression levels of *ACC*, *FAS*, and *SCD-1* were remarkably (*P* < 0.05) decreased, and the expression of lipid oxidation and bile acid metabolism genes *CPT-1*, *LXRα*, *CYP7A1*, and *CYP27A1* was enhanced in the 0.75% ATX group compared with the HFD group. These results indicated that consumption of a HFD contributed to fat synthesis and ultimately disturbed lipid metabolism; furthermore, high-dose ATX could improve the disorder of lipid metabolism by promoting cholesterol metabolism and inhibiting fat synthesis.

To explore how the hepatic lipidome is altered upon ATX intervention, RNA sequencing was used to accurately and quantitatively analyse liver transcriptional changes and lipid metabolism pathways in the liver in response to ATX supplementation. A total of 141 genes were differentially expressed in HFD-induced liver samples compared with ND-induced liver samples (Supplementary Figures 2A,B). However, a total of 156 differentially expressed genes, of which 103 were increased and 53 were decreased, were identified in the 0.75% ATX group compared with the HFD group (Supplementary Figures 2C,D). Additional screening revealed that 17 genes altered a multitude of biological processes related to the metabolic regulation of lipids, including fatty acid biosynthesis, cholesterol metabolism, lipid storage and bile acid metabolism, through the AMPK/SREBP1c signal pathway (Figures 4B,C).

### Astaxanthin Supplementation Altered Liver Lipid Metabolism by Lipidomic Analysis

**FIGURE 5:**
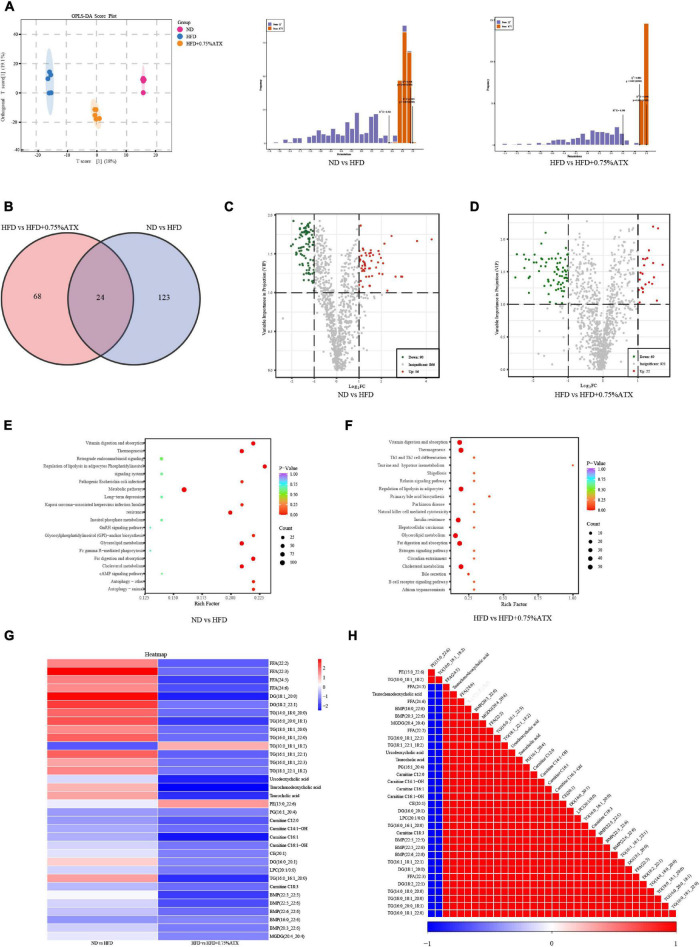
Astaxanthin regulated lipid metabolites in HFD-fed mice. **(A)** OPLS-DA score plot (*left*) and permutation plot (*right*). **(B)** Venn diagram depicting the overlap of significantly changed metabolites between experimental groups. The volcano plot analysis of ND vs. HFD group **(C)** and HFD vs. HFD + 0.75% ATX group **(D)**. Analysis of lipid metabolism pathway of ND vs. HFD **(E)** and HFD vs. HFD + 0.75% ATX **(F)**. **(G)** Heatmap of 34 significantly altered metabolites in ATX-treated HFD-fed mice. Blue: downregulated metabolites. Red: upregulated metabolites. **(H)** The associated heatmap of significantly changed metabolites.

We performed a comprehensive hepatic lipidomic analysis to evaluate whether differences in lipid content or composition may account for differences in hepatic lipid disorders between the HFD group and ATX group. The results of OPLS-DA comparison analysis showed a remarkable separation among the ND, HFD, and HFD + 0.75% ATX groups, indicating that HFD supplementation with ATX altered the compositions of liver lipids (Figure 5A). For the liver samples, the validation parameters of fitness (R^2^Y = 0.995 and Q^2^ = 0.954) were obtained in ND vs. HFD, as well as fitness (R^2^Y = 0.998 and Q^2^ = 0.882) in HFD vs. HFD + 0.75% ATX. A total of 1,012 lipid species were identified in liver samples, which belong to six primary classes of lipids, including glycerophospholipids (GPs), glycerides (GLs), fatty acyls (FAs), sphingolipids (SLs), sterol lipids (STs), and prenol lipids (PRs) (Supplementary Figure 3). Values with fold change ≥ 2 (fold change ≤ 0.5) and VIP > 1 were selected to represent metabolic biomarkers, and significant differences were identified at *P* < 0.05. Based on the abovementioned results, we screened 146 and 91 lipid biomarker candidates by applying volcano plots for such distinctions in ND vs. HFD and HFD vs. HFD + 0.75% ATX, respectively (Figures 5C,D).

According to the Venn diagram, we found that the accumulated 146 lipid species were significantly different between the ND and HFD groups, while ATX intervention patently changed the levels of 91 lipid species, including 24 ordinary species, compared to the levels in HFD-fed alone (Figure 5B). As shown in the heatmap, the HFD increased the levels of 13 metabolites (4 FFAs, 7 TGs, and 2 DGs) and decreased the levels of one metabolite (TG), whereas all of the metabolites were significantly inverted in the HFD + 0.75% ATX group (Figure 5G). Furthermore, in our present study, we found that 8 of the other 20 most relevant metabolites (3 BAs, 2 CARs, 2 BMP, and 1 TG) were remarkably downregulated after ATX supplementation; however, there was no significant difference in the ND vs. HFD group. We observed a significantly positive correlation among these 34 metabolite levels associated with lipid metabolism (Figure 5H). Thus, these results indicated that the 22 metabolites, including 4 FFAs, 8 TGs, 2 DGs, 3 BAs, 2 CARs, and 2 BMPs, might be potential biomarkers accountable for alleviating the steatohepatitis induced by lipid disturbance. The KEGG database was used to perform pathway analysis of differentially expressed metabolites. The pathways were considerably disrupted in the HFD group, including glycerolipid metabolism, insulin resistance, cholesterol metabolism, fat digestion and absorption, and regulation of lipolysis in adipocytes, when compared with the ND group; however, 0.75% ATX supplementation effectively prevented dysregulation of these pathways and improved bile secretion and primary bile acid biosynthesis (Figures 5E,F).

### Astaxanthin Supplementation Changed the Structures and Dysbacteriosis of Gut Microbiota in High-Fat Diet-Fed Mice

**FIGURE 6:**
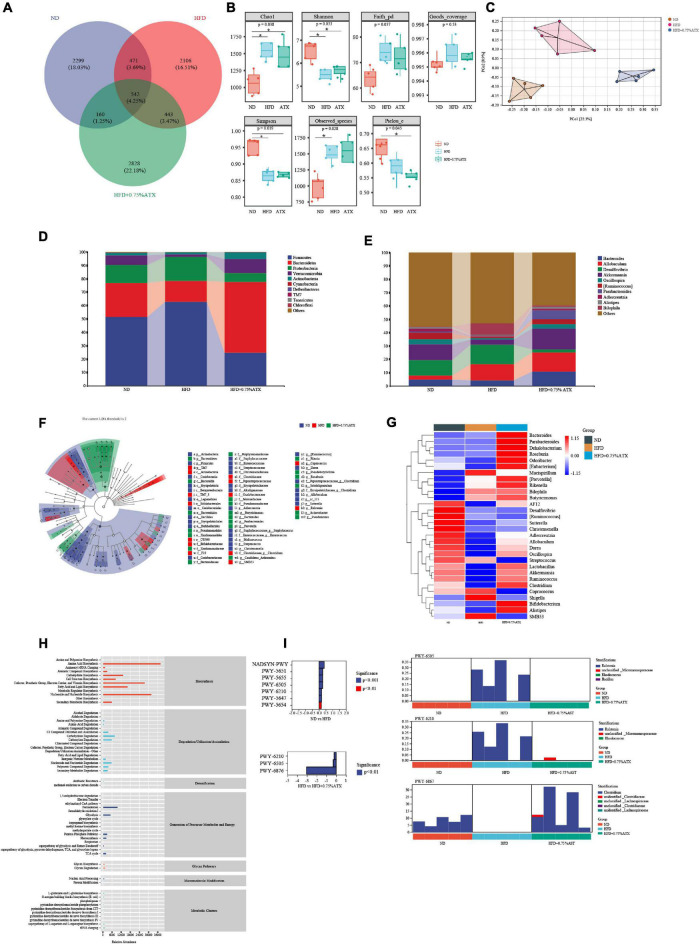
Astaxanthin regulated the gut microbiota. **(A)** The Venn diagram. **(B)** Evaluation of Alpha Diversity Index including Chao1, Simpson, Shannon, Pielou’s evenness, observed species, Faith’s phylogenetic diversity and Good’s coverage index values. Data were analyzed using a one-way ANOVA and are expressed as the mean ± SD. (*n* = 5). “*” *P < 0.05* versus ATX-treated HFD-fed mice. **(C)** PCoA of unweighted UniFrac distance from beta diversity analysis. **(D)** Phylum abundance graph genus levels. **(E)** Genus abundance graph. **(F)** Species taxonomy branch map based on LEfSe analysis. **(G)** The heatmap of the 30 bacterial genera with the largest differences in abundance were selected, according to the unweighted UniFrac distance of the intestinal content samples. **(H)** Predicted the abundance map of MetaCyc secondary functional pathways. X-coordinate: the abundance of functional pathways, Y-coordinate: the MetaCyc secondary functional pathway. **(I)** Analysis of differences in metabolic pathways (*left*) and species composition in different MetaCyc pathways (*right*).

To investigate how ATX prevents the progression and development of obesity and NAFLD by affecting the gut microbiota, we analyzed the 16S rRNA results clustered into different operational taxonomic units (OTUs) at a 97% similarity level. Of the 8,849 OTUs visualized in the experimental groups, 542 (4.25%) were normal among the ND, HFD, and 0.75% ATX groups. In addition, the number of other OTUs in the ND group, HFD group and 0.75% ATX group was 2,299 (18.03), 2,109 (16.51%), 2,828 (22.18%), respectively, indicating the great differences of gut microbes in the bacterial community (Figure 6A). Alpha-diversity analysis was used to identify the within-habitat diversity, including richness (the values of Chao1 and observed species), diversity (Shannon and Simpson), and evenness (Pielous’ evenness index), of which the richness significantly increased in the HFD and 0.75% ATX groups compared to the ND group; however, the Shannon and Simpson index decreased. The Goods coverage values had no obvious differences in each group (Figure 6B). To assess community similarity among samples, we applied principal coordinates analysis (PCoA) to represent the relative abundance of OTUs in each community by two different analyses. The PCoA plot showed that the structure and compositions of gut microbiota in the HFD group (Axis 1, 22.3%, Figure 6C) statistically separated from the ND group, and a considerable distinction was observed in the 0.75% ATX group (Axis 2, 8.9%).

At the phylum level, the taxonomic profiles of the gut microbiomes showed significant differences according to increasing ATX supplementation and developing obesity severity, within which *Firmicutes*, *Bacteroidetes*, and *Proteobacteria* were the dominant phyla. The abundance of *Firmicutes*, *Proteobacteria*, *Verrucomicrobia*, and *Actinobacteria* decreased significantly, and the proportions of *Bacteroidetes* increased markedly (*P* < 0.05) in the HFD group compared with those of the ND group (Figure 6D), while 0.75% ATX supplementation further reduced the abundance of *Firmicutes* and *Proteobacteria* and remarkably reversed the trend of the other three bacteria. At the genus level, the abundance of genera, including *Bacteroides*, *Allobaculum*, *Desulfovibrio*, *Akkermansia*, *Oscillospira*, *Ruminococcus*, *Parabacteroides*, *Adlercreutzia*, *Alistipes*, and *Bilophila*, was significantly altered by a high-fat diet compared with the normal diet and moderately inverted by 0.75% ATX. Compared to the mice induced by HFD alone, the mice supplemented with ATX had significantly upregulated abundances of *Akkermansia* and *Parabacteroides* to 15.74 and 6.73%, respectively (Figure 6E). Additionally, to explore high-dimensional biomarkers and identify significant differences at the species level, LEfSe with default parameters was used between the microbial communities compared. The 65 most abundant OTUs were observed at the taxonomic level in the samples, among which beneficial bacteria were significantly reduced in the HFD group compared with the ND group, revealing a serious gut microbial disorder in HFD-fed mice (Figure 6F).

Furthermore, 9 of the 30 most prevalent bacterial genera were upregulated and 21 bacterial genera were downregulated in the HFD-fed mice compared with the mice fed a normal diet, while these genera were partially promoted to their original relative abundance levels after ATX supplementation (Figure 6G). To characterize the functional role of the related abundant bacterial genera, we found 47 secondary functional pathways from the MetaCyc database of metabolic pathways that are relevant to lipometabolism, including the fatty acid and lipid biosynthesis pathway (abundance value: 16,383.26), carbohydrate degradation (8,059.48), secondary metabolite biosynthesis (6,077.67), glycolysis (5,101.02), secondary metabolite degradation (3,331.28), and pentose phosphate pathways (2,978.49) (Figure 6H). According to the MetaCyc profiles, there were significant and apparent differences in the species composition of the eight selected metabolic pathways, among which three pathways, 2-aminophenol degradation (PWY-6210), L-tryptophan degradation XII (PWY-6505) and isopropanol biosynthesis (PWY-6876), were relatively and significantly (*P* < 0.05) different in all of the samples (Figure 6I).

## Discussion

Obesity and obesity-related complications are classic health problems worldwide. A long-term high-fat diet and an imbalance in energy expenditure are important causes for concern (26). In both obese individuals and animal models of NASH, it could be characterized by excessive intracellular lipid accumulation combined with inflammation, which can ultimately progress into hepatic insulin resistance, mitochondrial dysfunction and cellular injury (27, 28). Emerging evidence shows that ATX, a natural functional food, has been used as a dietary supplement for treating obesity and liver injury and maintaining health (18, 29). For example, Ni’s study reported that ATX reduced hepatic steatosis and TG accumulation by decreasing lipid uptake and further improved simple fatty liver whether in a HFD-induced obesity (DIO) model or in genetically obese (*ob*/*ob*) mice (30). Importantly, when compared to vitamin E, ATX was more effective at lipid peroxidation and preventing NASH. In the present study, our results showed that ATX supplementation could prevent obesity and the development of NAFLD by meditating lipid metabolism and gut microbiota. Alternatively, ATX consumption also prevents oxidative stress in the liver and lipid peroxidation by improving antioxidant enzyme activity. According to experimental results, dietary ATX not only significantly decreased body weight gain, adipose tissue weight, and serum TG, TC, and LDL-C levels but also ameliorated abnormal hepatic metabolism following the reduction of liver weight and hepatic TG and TC levels in HFD-induced mice. No significant difference in the food efficiency ratio or serum HDL-C levels was observed in the HFD group with long-term ATX intake. From the physiological and biochemical profiles, ATX exhibited a better preventive effect on dyslipidaemia and abnormal liver function than our previous results (18).

Over the past decade, numerous pieces of evidence have shown that oxidative stress caused by a high-fat diet and specific products of ROS are involved in the development of obesity and fatty liver (31, 32). Thus, balancing the liver oxidative reaction is an important aspect of preventing the development of NAFLD. Studies have shown that oxidative stress is closely related to endoplasmic reticulum (ER) stress in the development and progression of NAFLD and other diseases, while ATX can directly or indirectly moderate ER through antioxidant activity (33, 34). Interestingly, previous study has confirmed that ATX significantly reduced the levels of oxidative stress marker thiobarbituric acid-responsive substances (TBARS) in the liver of NASH mice (30). In our results, both the ROS levels evaluated by the DHE probe and the levels of MDA measured, a lipid peroxidation product, were significantly increased in liver tissues in each experimental group. HFD might have contributed to the increase in these oxidative stress indices and the decrease in antioxidant enzymes, including T-AOC, SOD, CAT, and GSH levels. Our results are consistent with previous studies showing that HFD seriously damaged the antioxidant defense system (32, 35). Regardless of the dose, the MDA levels of all ATX-supplemented groups were reduced, suggesting that ATX suppresses overproduction of ROS induced by obesity. In addition, with dose-dependent increases of the ATX in the diet, the activities of antioxidant enzymes remarkedly improved and were close to normal levels in mice fed HFD. Multiple studies have confirmed that cell apoptosis induced by excessive endogenous cholesterol is associated with increased ROS in tissues (36, 37). As previously discussed, long-term HFD intake advanced total cholesterol and disturbed the oxidative balance in the liver, which was attributed to hepatocellular apoptosis. Based on the TUNEL assay results, we found a large number of apoptotic liver cells in the HFD group, whereas ATX alleviated the degree of necrosis. Nevertheless, the precise intracellular mechanism responsible for this phenomenon was unclear in this study. Moreover, the pathological results showed that ATX could effectively prevent fat accumulation and hepatic steatosis in a dose-dependent manner.

Whether for obesity or the development of NAFLD, one of the root causes is the perturbation in lipid metabolism (38). As reported in previous studies, excessive fat intake induced abnormal bile secretion and disturbed cholesterol levels (39). In addition, FFAs usually trigger the accumulation of DGs and TGs by mediating insulin signal and sensitivity in liver tissue (40). To demonstrate the function of ATX in lipid metabolism, lipidomic analysis revealed that the total levels of hepatic FFAs, TGs, and DGs were noticeably increased in HFD group mice, indicating that a high-fat diet partly supported our previous results. Interestingly, our results suggested that ATX not only decreased the levels of FFAs and TGs but also specifically reduced the levels of BAs and acyl-carnitines, indicating that both cholesterol metabolism and fatty acid oxidation were improved in mouse livers. Moreover, *SREBP1c*, along with its downstream genes *ACC*, *SCD1* and *FAS*, is an important component in the energy metabolic system and plays a key role in regulating the FFA and TG synthesis mentioned above (38, 41).

To further clarify the association between the development of obesity and the changes in lipid metabolites and related genes, we integrated the transcriptome and qPCR analysis to verify the gene expression levels in the AMPK/SREBP1c pathway. According to transcriptome analysis, gene expression signatures were profoundly distinguished among the experimental groups. Considering the degree and diversity of gene expression changes, only genes associated with the target pathway were screened in this study. *AMPK*, a key molecule in the regulation of biological energy metabolism, is involved in diabetes and metabolism-related diseases (42). Peroxisome proliferator activated receptor (*PPARα*) and peroxisome proliferator-activated receptor gamma coactivator-1α (*PGC-1*) play an important role in regulating the homeostasis of adipose tissue by jointly regulating the balance between fatty acid synthesis and oxidation (43). The expression of *PPARα*, which is negatively correlated with the severity of NASH, is significantly reduced in NAFLD (44). ATX alleviated the gene expression associated with EIF-2 signaling in NASH rather than improved the expression of gene related to mitochondrial dysfunction (45). In present study, the results revealed that dietary 0.75% ATX significantly decreased the expression levels of *AMPK*, *SREBP1c*, *ACC*, *FAS*, *PPARγ*, and *SCD-1* while increasing the expression levels of *PPARα*. As our previous manuscript shown, the interaction between *PPARα* and *PGC-1α* promoted the oxidation of fatty acids and inhibited the expression of *SREBP1c* to a certain extent (18). During lipid metabolism, *CPT-1* is a key rate-limiting enzyme that accelerates the entry and β-oxidation of long-chain fatty acids into mitochondria (46). A high-fat diet suppresses the expression of *PGC-1α*, and the mitochondrial respiration rate decreases in the absence of *PGC-1α*, ultimately leading to a decrease in fatty acid oxidation capacity. In present study, our results found 0.75% ATX effectively up-regulated the expression level of *PGC-1α* and further stimulated the expression of its downstream gene *CPT-1α*, indicating that ATX reduced intracellular lipid content by promoting fatty acid oxidation. A previous study confirmed that the suppression of *SCD-1* could effectively attenuate HFD-induced insulin resistance and hepatic steatosis (47). However, we did not examine the effects of *SCD-1* knockdown or overexpression on liver lipid metabolism in mice, which should be addressed in future studies. Cholesterol 7α-hydroxylase (*CYP7A1*) and cytochrome P450 27A1 (*CYP27A1*), two important rate-limiting enzymes, play major roles in maintaining the balance of cholesterol and bile acid in the bile acid biosynthetic pathway in the liver (48). The decrease in serum cholesterol is due to a decrease in its de novo synthesis in the liver and an increase in the conversion to bile acids. *LXRα*, as nuclear receptors, regulates the transcription of *CYP7A1*, which is related to regulation of cholesterol and bile acids metabolisms (49). Our data showed that a HFD could downregulate *LXRα*, *CYP7A1*, and *CYP27A1* expression, while high-dose ATX supplementation could upregulate them, indicating that ATX could eliminate excess cholesterol in liver tissue by stimulating the conversion of cholesterol to bile acids. Besides, the expression of *CYP7A1* and *CYP27A1* is regulated by the intestinal flora (50). In summary, our study provides evidence to support the hypothesis that ATX may mediate cholesterol metabolism, fatty acid oxidation and synthesis via the AMPK/SREBP1c pathway to prevent obesity and fatty liver.

The characteristic role of diet in obesity and metabolic disorders is that diet has become an important factor in regulating the gut environment. Both long-term and short-term dietary interventions will induce changes in the structure and function of intestinal microbes (51). Briefly, liver metabolites mainly affect the composition of gut microbes and the integrity of the intestinal barrier, while gut microbiota regulate the synthesis of bile acids, glucose and lipid metabolism in the liver (52). Noticeably, many recent studies have shown that functional foods and natural health products, directly or indirectly, prevent obesity and metabolic diseases by improving intestinal diversity (25, 53). In the current study, mice fed a HFD exhibited lower diversity and gut microbiota disturbance; however, ATX had a salutary effect on promoting gut microbiota and improving diversity. ATX significantly reduced the values of *Firmicutes/Bacteroidetes* (F/B), indicating that the reduction in weight gain might be partly due to changes in F/B. Moreover, the increase in *Actinobacteria* and *Verrucomicrobia* at the phylum level indicated that ATX effectively activated functional bacteria.

Astaxanthin treatment had the greatest impact on *Allobaculum* and *Akkermansia* at the genus level. The relative abundance of *Allobaculum* is associated with hormone secretion, SCFA production, serum HDL-C concentration, and intestinal barrier integrity, which is usually found in higher relative abundances in healthy individuals in prior studies (54, 55). In addition, *Akkermansia*, an intestinal symbiont colonizing the mucosal layer, is considered to be a functional probiotic that is closely related to fat increase, secondary bile acid biosynthesis and IR (56). In this study, the relative abundance of *Allobaculum* and *Akkermansia* in the 0.75% ATX group was even higher than that in the ND group. In addition, ATX significantly decreased the abundance of *Desulfovibrio*, a pathogenic bacterium that induces lipopolysaccharide (57). *Lactobacillus*, *Clostridum*, and *Bifidobacterium* are closely associated with cholesterol metabolism, and the abundance of *Bifidobacterium* is positively correlated with the level of high-density lipoprotein (HDL) (58). Surprisingly, further screening found that ATX, in contrast to chalk and cheese from our previous research, promoted *Butyricimonas*, *Lactobacillus*, *Clostridum*, and *Bifidobacterium* in the current results. As the results shown, 0.75% ATX significantly increased the abundance levels of *Lactobacillus*, *Clostridum*, and *Bifidobacterium*, implying that these probiotics may directly or indirectly regulate the expression of genes related to bile acid synthesis, which was consistent with previous report (49). Another interesting finding in this study was the promotion of *Butyricimonas*, a probiotic that produces butyric acid metabolites, after ATX supplementation, which contributed to alleviating systemic obesity and gut inflammation. Moreover, butyrate can restore intestinal mucosal injury induced by a high-fat diet and reduce nonalcoholic steatohepatitis (59). Thus, according to the increase in these functional probiotics, ATX supplementation could effectively prevent the microbial dysbiosis induced by HFD. All in all, when compared to our previous study, we found that the preventive effect of ATX is better than its therapeutic effect whether from the physiological and biochemical level, from the metabolic level or from the multi-omics and pathological level.

In conclusion, the current study shows that ATX has a better preventive effect on the development of obesity and NAFLD induced by HFD when compared with prognosis treatment. Both physiological and biochemical profiles demonstrate that long-term consumption of ATX effectively prevents body weight gain, dyslipidaemia and abnormal liver function. Subsequently, pathological analysis indicated that ATX relieves liver steatosis, as well as oxidative stress and apoptosis caused by excessive fatty acids. In addition, we also confirmed that ATX regulates lipid metabolism by mediating the expression of genes in the AMPK/SREBP1c pathway. Moreover, ATX improves hepatic lipid metabolism and increases gut probiotics, confirming that some metabolites might be positively correlated with specific bacteria to maintain body health through the liver-gut axis. This study provides scientific evidence for the functional effects of ATX on obesity prevention. However, our research still has some limitations. Future studies should focus on verifying the role of different pathways in the regulation of lipid metabolism at the protein level and exploring the molecular mechanism of liver oxidative stress and apoptosis through fatty acids mediated by ATX.

## Data Availability Statement

The data presented in the study are deposited in the DRYAD repository, accession number: doi: 10.5061/dryad.dz08kprzs.

## Ethics Statement

The animal study was reviewed and approved by Institutional Animal Care and Use Committee of Shanxi Agricultural University.

## Author Contributions

MW and WX: conceptualization and methodology. MW, WX, JY, YL, CJ, CZ, JX, and RL: validation and investigation. MW: formal analysis, writing—original draft preparation, visualization, and project administration. HC: resources, writing—review, editing, supervision, and funding acquisition. All authors have read and agreed to the published version of the manuscript.

## Conflict of Interest

The authors declare that the research was conducted in the absence of any commercial or financial relationships that could be construed as a potential conflict of interest.

## Publisher’s Note

All claims expressed in this article are solely those of the authors and do not necessarily represent those of their affiliated organizations, or those of the publisher, the editors and the reviewers. Any product that may be evaluated in this article, or claim that may be made by its manufacturer, is not guaranteed or endorsed by the publisher.

## Supplementary Material

Supplementary Figure 1 Evaluation of TUNEL reagent on cell apoptosis. (A) The positive result of is indicated by the green marked spots in the sample. (B) Apoptosis rate of each treatment. Values are expressed as mean ± SD of triplicate. The different letters (a–d) indicate significant differences (*P* < 0.05) according to Duncan’s multiple range test in ANOVA.

Supplementary Figure 2 The screened genes and Volcano plot of ND vs. HFD and HFD vs. HFD + 0.75% ATX by RNA-seq results.

Supplementary Figure 3 Cluster heatmap of metabolites in all samples.
