# Supplementary material for: Astaxanthin From Haematococcus pluvialis Prevents High-Fat Diet-Induced Hepatic Steatosis and Oxidative Stress in Mice by Gut-Liver Axis Modulating Properties
Source: Front Nutr. 2022 Apr 12;9:840648. doi: 10.3389/fnut.2022.840648 (PMC9039660; doi:10.3389/fnut.2022.840648)
Supplement: Supplementary file 1 [file Data_Sheet_1.PDF]

Table S1. The composition of experimental diet

Ingredient	ND/g	HFD/g
casein	189.58	233.06
L-cystine	2.84	3.5
Cornstarch	298.59	84.83
Maltodextrin	33.18	116.53
Sucrose	331.77	201.36
Cellulose	47.4	58.26
Soybean oil	23.7	29.13
Lard	18.96	206.84
M1002 mineral mix	9.48	11.65
Calcium hydrogenphosphate	12.32	15.15
Calcium carbonate	5.21	6.41
Patassium citrate	15.64	19.23
V1001 vitamin mixture	9.48	11.56
Choline bitartrate	1.9	1.9
Food dye	0.047	0.058
Total	1000	1000

Table S2. Primer sequences used for qRT-PCR

Target Gene	Primers
GAPDH	5'-AGGTCGGTGTGAACGGATTTG-3'
	5'-TGTAGACCATGTAGTTGAGGTCA-3'
AMPK	5'-AGTGTTCGGAGGAGGAGGT-3'
	5'-TGTCTATGATGAGGTGGTAGGC-3'
SREBP1c	5'-TTCAGAAGTTGCGGTCACAC-3'
	5'-CTGGTTGCTCTGCTGAAG-3'
PPARα	5'-TCCACGAAGCCTACCTGAAG-3'
	5'-GAATCGGACCTCTGCCTCTT-3'
PPARγ	5'-GATGGAAGACCACTCGCATTC-3'
	5'-CCACAGACTCGGCACTCAA-3'
FAS	5'-AAGGAGTACATGGACAAGAACC-3'
	5'-CAGGAGAATCGCAGTAGAAGTC-3'
SCD-1	5'-GAGGAGAGGAAGGAAGGGAAG-3'
	5'-GATGCCAGGAGAGCCAAGA-3'
LXRα	5'-ATTCTTCCGCCGCAGTGT-3'
	5'-AGGACTTGAGGAGGTGAGGA-3’
CPT-1	5'-CAACACCATCCACGCCATAC-3’

	5'-GAGCCAGACCTTGAAGTAACG-3’
CYP7A1	5'-GCCTTCTGCTACCGAGTGAT-3’
	5'-AGGTGGAGAGTGTATCGTTGAG-3’
CYP27A1	5'-GAGCAAGTGATGAGACAGGAG-3’
	5'-GCAAGGTGGTAGAGAAGATGAG-3’
ACC-1	5'-TCGCCTGACAACAACTGGAA-3’
	5'-GACTGTGCCTGGAACCTCTT-3’
PGC-1	5'-ATCAAGGTCTCCAGGCAGTAG-3’
	5'-GCATCACAGGTATAACGGTAGG-3’
