# Supplementary figures and images for: Astaxanthin From Haematococcus pluvialis Prevents High-Fat Diet-Induced Hepatic Steatosis and Oxidative Stress in Mice by Gut-Liver Axis Modulating Properties
Source: Front Nutr. 2022 Apr 12;9:840648. doi: 10.3389/fnut.2022.840648 (PMC9039660; doi:10.3389/fnut.2022.840648)

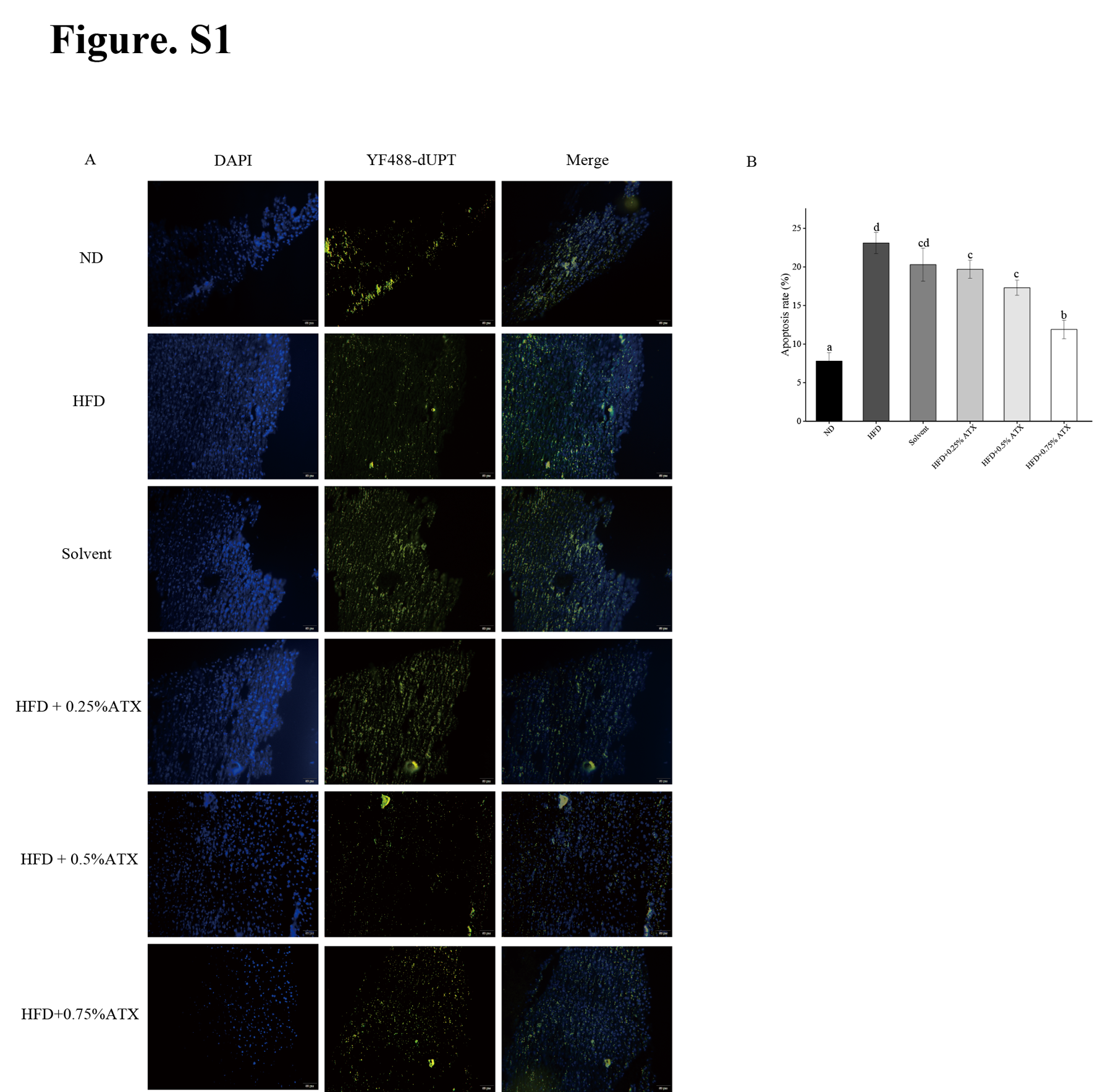

Supplement: Supplementary Figure 1 — Evaluation of TUNEL reagent on cell apoptosis. (A) The positive result of is indicated by the green marked spots in the sample. (B) Apoptosis rate of each treatment. Values are expressed as mean ± SD of triplicate. The different letters (a–d) indicate significant differences (P < 0.05) according to Duncan’s multiple range test in ANOVA. [file Image_1.TIF]

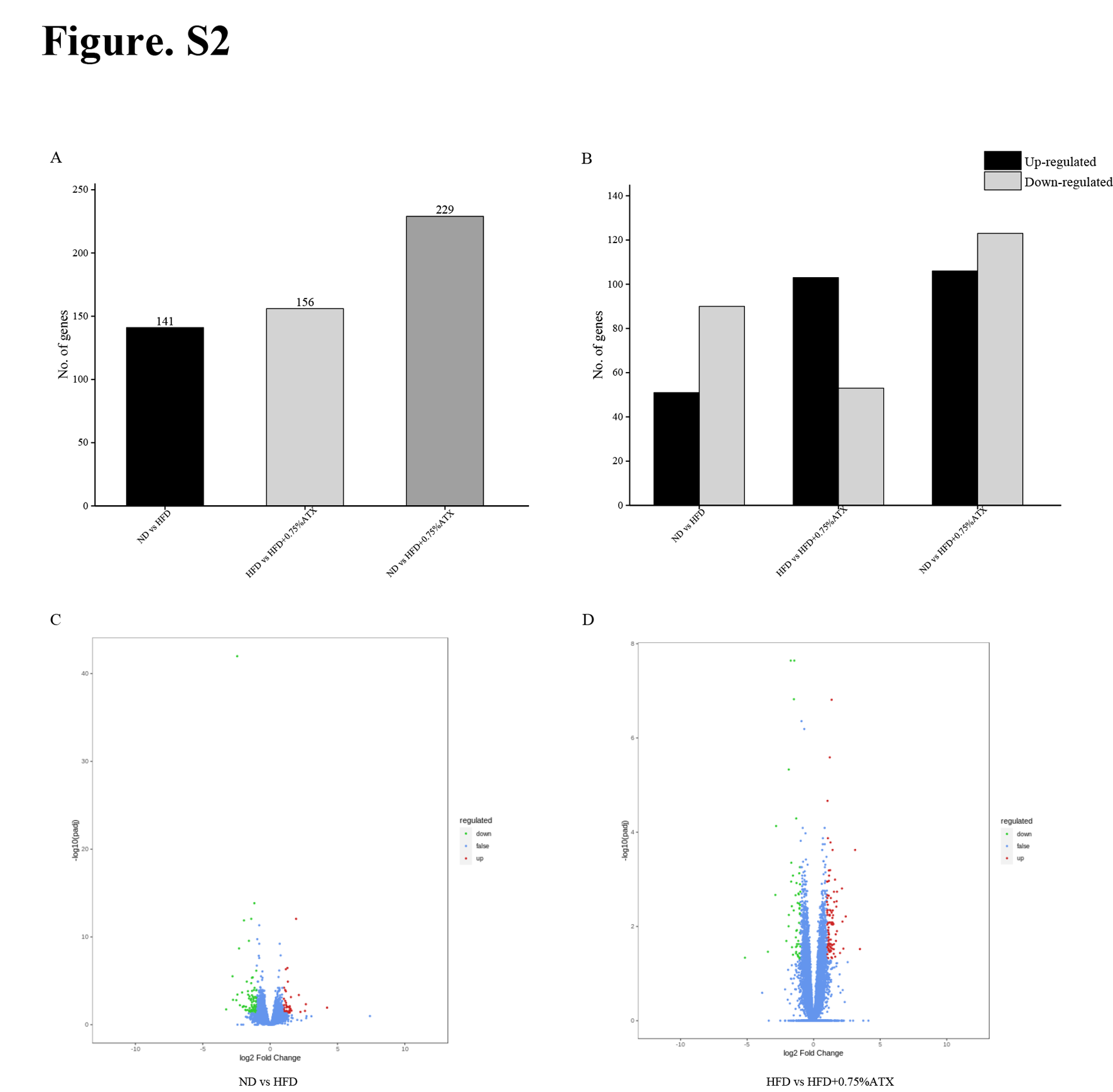

Supplement: Supplementary Figure 2 — The screened genes and Volcano plot of ND vs. HFD and HFD vs. HFD + 0.75% ATX by RNA-seq results. [file Image_2.TIF]

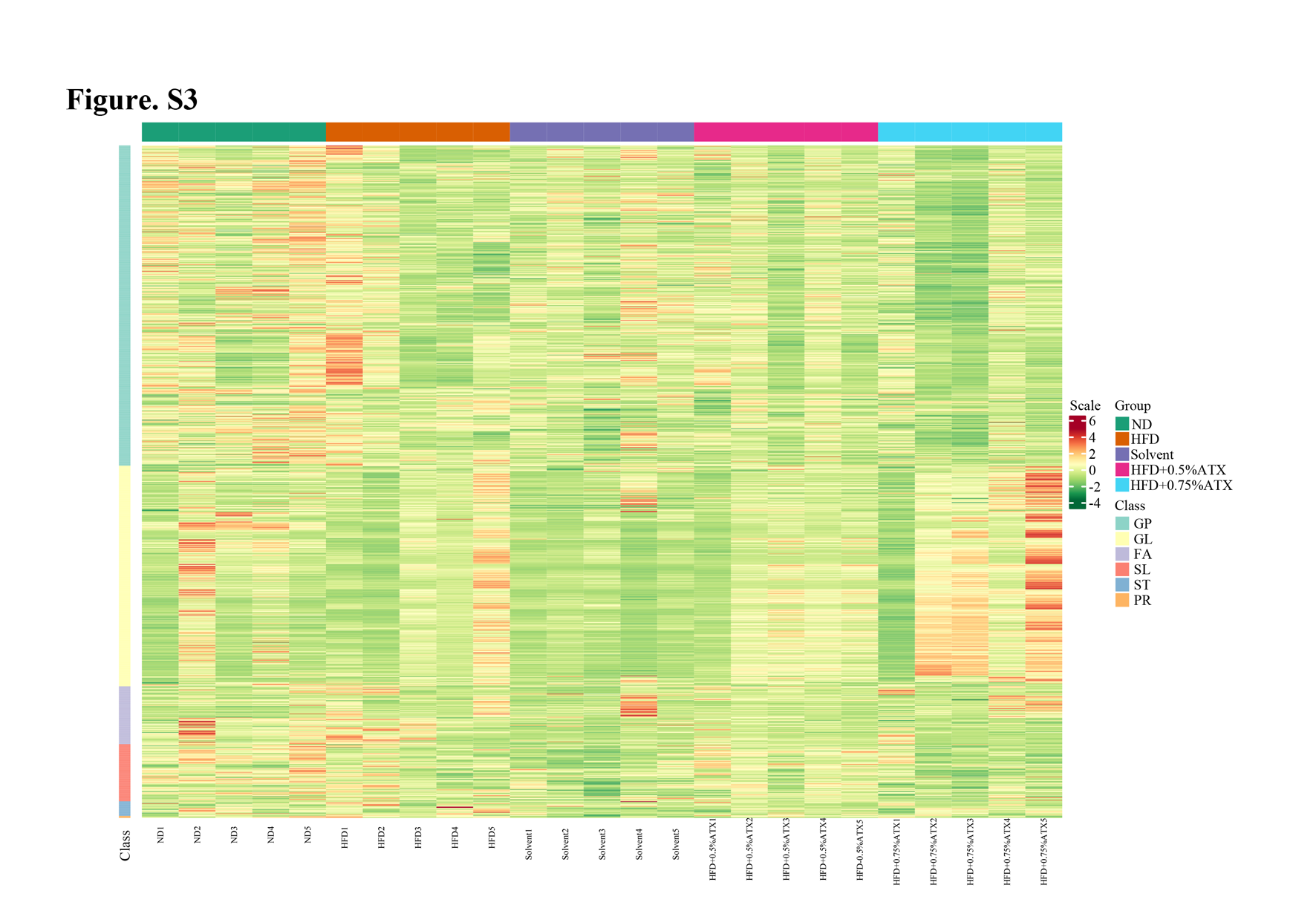

Supplement: Supplementary Figure 3 — Cluster heatmap of metabolites in all samples. [file Image_3.TIF]
